# Clinical usefulness of digital twin guided virtual amiodarone test in patients with atrial fibrillation ablation

**DOI:** 10.1038/s41746-024-01298-z

**Published:** 2024-10-23

**Authors:** Taehyun Hwang, Byounghyun Lim, Oh-Seok Kwon, Moon-Hyun Kim, Daehoon Kim, Je-Wook Park, Hee Tae Yu, Tae-Hoon Kim, Jae-Sun Uhm, Boyoung Joung, Moon-Hyoung Lee, Chun Hwang, Hui-Nam Pak

**Affiliations:** 1 Division of Cardiology, Department of Internal Medicine, Yonsei University College of Medicine, Seoul, Republic of Korea; 2 Division of Cardiology, Department of Internal Medicine, Yongin Severance Hospital, Yonsei University College of Medicine, Yongin, Republic of Korea

**Keywords:** Atrial fibrillation, Atrial fibrillation, Cardiovascular models, Computational models

## Abstract

It would be clinically valuable if the efficacy of antiarrhythmic drugs could be simulated in advance. We developed a digital twin to predict amiodarone efficacy in high-risk atrial fibrillation (AF) patients post-ablation. Virtual left atrium models were created from computed tomography and electroanatomical maps to simulate AF and evaluate its response to varying amiodarone concentrations. As the amiodarone concentration increased in the virtual setting, action potential duration lengthened, peak upstroke velocities decreased, and virtual AF termination became more frequent. Patients were classified into effective (those with virtually terminated AF at therapeutic doses) and ineffective groups. The one-year clinical outcomes after AF ablation showed significantly better results in the effective group compared to the ineffective group, with AF recurrence rates of 20.8% vs. 45.1% (log-rank p = 0.031, adjusted hazard ratio, 0.37 [0.14-0.98]; p = 0.046). This study highlights the potential of a digital twin-guided approach in predicting amiodarone’s effectiveness and improving personalized AF management. Clinical Trial Registration Name: The Evaluation for Prognostic Factors After Catheter Ablation of Atrial Fibrillation: Cohort Study, Registration number: NCT02138695. The date of registration: 2014-05. URL: https://www.clinicaltrials.gov; Unique identifier: NCT02138695.

## Introduction

Active rhythm control for patients with atrial fibrillation (AF) has been recommended since the Early Treatment of Atrial Fibrillation for Stroke Prevention Trial (EAST-AFNET4 trial), and antiarrhythmic drugs (AADs) are essential first-line therapies to improve survival and quality of life^1^. However, current AF management guidelines prioritize the safety of AADs over their efficacy^2^. This emphasis on safety is largely influenced by the findings of the AFFIRM trial^3^, which demonstrated that rhythm control with AADs did not offer a survival advantage over rate control, raising concerns about potential adverse drug effects. While guideline-based AF management has successfully reduced the risk of side effects to just 4.9%^1^, the EAST-AFNET4 trial does not provide specific recommendations regarding the optimal dosage or effectiveness of these drugs. Consequently, a cautious, safety-first approach to AAD use may inadvertently compromise their efficacy. Therefore, there is a clear need to reassess AAD efficacy while maintaining the safety improvements achieved in current practices. Although the mechanisms of AADs have been challenging to elucidate due to the complexities of the disease and the drugs themselves^4,5^, recent advances using the digital twin of the human heart have made it possible to suggest potential mechanisms of AF and screen for new AADs by changing the ion currents^6–8^. A digital twin is a dynamic virtual replica of a physical entity that simulates its behavior and interactions within a system^9^. Various types of digital twins of the heart have been introduced, including those integrating patients’ magnetic resonance imaging (MRI) data^10–12^.

In this study, the digital twin was created using computed tomography (CT) and electroanatomical mapping (EAM) data of the left atrium (LA) from patients with AF^13^. Through mathematical and computational modeling, a virtual LA was constructed to induce and observe virtual AF, with the aim of advancing personalized medicine. We previously reported the accuracy and clinical usefulness of the AF digital twin for guiding appropriate AF catheter ablations^14–17^. Additionally, we reported the feasibility of AF digital twin for selecting appropriate AADs^18^ and explored the usefulness of the digital twin-guided virtual amiodarone (AMD) test^18–20^. Notably, this is the first study to evaluate the clinical outcomes of patients with AF using AMD after ablation by comparing clinical outcomes with the virtual AMD responses observed in the digital twin. This single-center retrospective study aimed to evaluate the clinical usefulness of the virtual AMD test by comparing the 1-year rhythm outcomes of AMD users with the virtual AMD test results using the digital twin, thereby laying the foundation for future prospective randomized studies.

## Results

### AF Digital twin

**Fig. 1:**
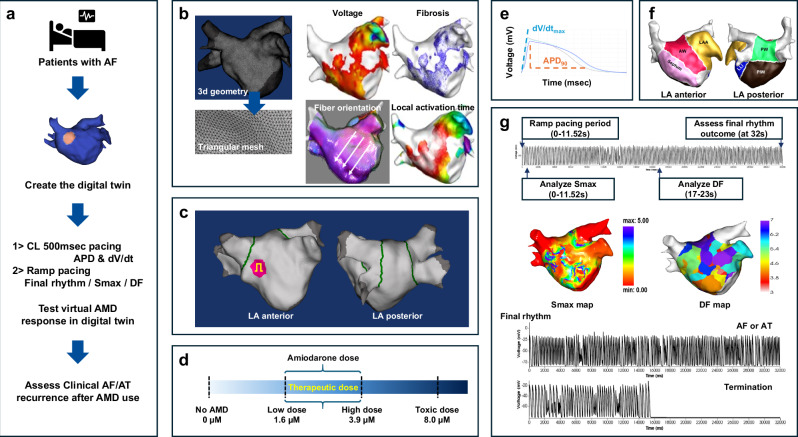
The scheme and process of creating an AF digital twin and conducting a virtual AMD test. **a** Creation of a patient-specific digital twin for patients with AF who underwent catheter ablation. **b** Construction of a 3D geometry with over 400,000 nodes arranged in a triangular mesh. Virtual maps were generated to characterize electrophysiological properties, including voltage, fibrosis, fiber orientation, and local activation time. **c** Virtual PVI procedure, with green lines representing ablation sites, pink areas indicating early activation sites, and yellow denoting pacing locations. **d** Virtual AMD testing under different conditions: no AMD (control), low dose, high dose, and toxic dose. **e** During constant 500 ms pacing across various AMD concentrations, surface EGMs were recorded, with time and voltage plotted to calculate human APD and dV/dt. **f** Segmentation method employed for the extra-pulmonary vein area of the LA. **g** Observation of AF post-ramp pacing over 32 seconds, with the creation of Smax maps using APD and diastolic interval, and DF maps during the maintenance phase of AF or AT between 17 and 23 seconds. EGMs show AF/AT maintenance until 32 seconds and termination before 16 seconds. AF atrial fibrillation, AMD amiodarone, AT atrial tachycardia, APD action potential duration, APD_90_ 90% of action potential duration, CL cycle length, DF dominant frequency, dV/dt: peak upstroke velocity, EGM electrogram, Smax the maximal restitution slope of action potential duration, LA left atrium, PVI pulmonary vein isolation.

We developed a patient-specific digital twin of the LA (Fig. 1a). To create this digital twin, we integrated CT imaging and EAM data obtained during AF catheter ablation (AFCA), characterizing electrophysiological and histological properties, including fibrosis, fiber orientation, and synchronization of the local activation time (LAT) map (Fig. 1b). After virtual pulmonary vein isolation (PVI) (Fig. 1c), we were able to induce virtual AF by adjusting multiple ion channel conductivities and simulating the effects of AMD by modifying the blockade of ion channels based on its concentration and Hill’s coefficient (Fig. 1d).

This study aimed to:

1. Analyze electrophysiological parameters, such as action potential duration (APD) and peak upstroke velocity (dV/dt), under constant 500 ms pacing conditions (Fig. 1e).
2. Assess virtual rhythm outcomes and wave dynamics parameters, including dominant frequency (DF)^21^ and the maximal slope of the APD restitution curve (Smax)^22^, during AF induction simulations (Fig. 1g).
3. Investigate whether the clinical outcomes of patients who received AMD after ablation are correlated with the virtual AMD response in the digital twin.
4. Analyze regional differences in Smax and DF based on AMD dosage or virtual rhythm outcomes through LA segmentation (Fig. 1f).

**Fig. 2:**
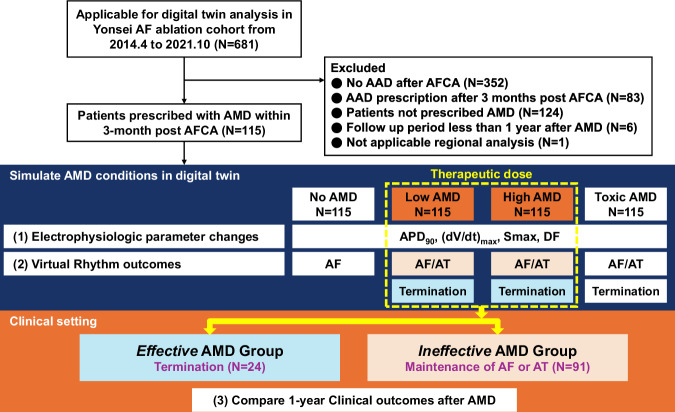
Flow chart of the study. We included a total of 115 patients and performed pacing at different AMD concentration settings. We observed changes in electrophysiological parameters and induced AF to assess virtual rhythm outcomes. AF termination at least once at any therapeutic dose was classified as *Effective*, while ongoing AF or AT was classified as *Ineffective*. Based on the results of virtual outcomes, we compared the clinical rhythm outcomes one year after clinical AMD usage. AAD antiarrhythmic drug, AF atrial fibrillation, AFCA atrial fibrillation catheter ablation, AMD amiodarone, APD_90_ 90% of action potential duration, AT atrial tachycardia, DF dominant frequency, Smax the maximal restitution slope of action potential duration.

This approach enabled us to better understand the relationship between digital simulations and real-world outcomes in AF management. This scheme is illustrated in Fig. 2.

### Electrophysiological changes with virtual AMD

**Fig. 3:**
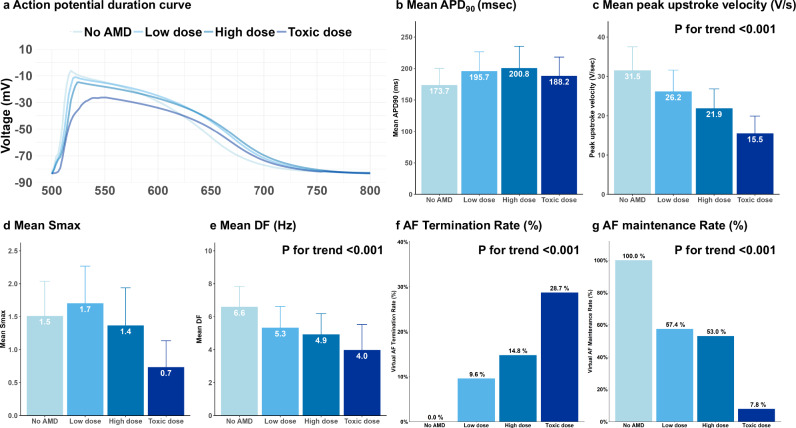
Effects of virtual AMD on electrophysiological parameters and virtual AF outcomes. **a** Action potential duration (APD) curves plotted under constant 500 ms pacing at various AMD concentrations. **b** A significant increase in APD_90_ is observed with both low and high doses of AMD, with a slight decrease at the toxic dose, although still higher than the baseline (No AMD). **c** The peak upstroke velocity decreases significantly in a dose-dependent manner as the concentration of AMD increases. **d** Low-dose AMD increases Smax compared to baseline, while high-dose and toxic-dose AMD significantly reduce Smax. **e** There is a significant reduction in DF as the dose of AMD increases, indicating decreased AF driver activity. **f** As the dose of AMD increases, the rate of AF termination increases, showing the drug’s effectiveness at higher concentrations. **g** The AF maintenance rate decreases significantly with increasing AMD concentration, reflecting the enhanced likelihood of AF termination at higher doses. AF atrial fibrillation, AMD amiodarone, AT atrial tachycardia, APD action potential duration, DF dominant frequency, dV/dt: peak upstroke velocity, Smax the maximal restitution slope of action potential duration.

In Fig. 3a, under constant 500 ms pacing, we plotted the APD curves across different AMD concentrations in the digital twin. The APD measured at 90% repolarization of the action potential amplitude (APD_90_) increased significantly under both low-dose (p < 0.001) and high-dose AMD (p < 0.001) conditions compared to the baseline (Fig. 3b). Although the APD_90_ decreased at toxic doses compared to low doses, it remained significantly higher than in the no- AMD condition (p < 0.001). Figure 3c shows that the peak upstroke velocity (dV/dt [V/s]) decreased significantly in a dose-dependent manner (p for trend <0.001).

### AF Wave-dynamic parameters under virtual AMD

In Fig. 1g, during ramp pacing for 11.52 seconds, we plotted the diastolic interval (DI) against the APD to generate the APD restitution curve. The Smax was calculated at each node, and the mean Smax was analyzed across various AMD concentrations. Low-dose AMD significantly increased Smax (p < 0.001 vs. baseline), while high-dose AMD reduced it (p = 0.016 vs. baseline; Fig. 3d and Supplementary Table 1). At the toxic dose, Smax was substantially decreased (p < 0.001). In Fig. 1g, during the 17 to 23 seconds following pacing, while AF or atrial tachycardia (AT) was stably maintained, the DF for all nodes was calculated using Fast Fourier Transform (FFT), and the mean values were compared. As shown in Fig. 3e, the mean DF decreased significantly in a dose-dependent manner as the AMD dose increased (p for trend <0.001).

### Effects of virtual AMD on induced virtual AF

In Fig. 1g, following ramp pacing, we evaluated the final rhythm of the digital twin, determining whether the outcome was sustained AF, regular AT, or termination with no detectable signal. As illustrated in Fig. 3f, g, in the absence of AMD, AF was maintained in 100% of cases. However, as AMD concentrations increased—from low to high and toxic doses—the AF termination rate progressively increased, while the maintenance rate correspondingly decreased (both p for trend <0.001).

### Clinical rhythm outcomes vs. virtual AMD Test

**Fig. 4:**
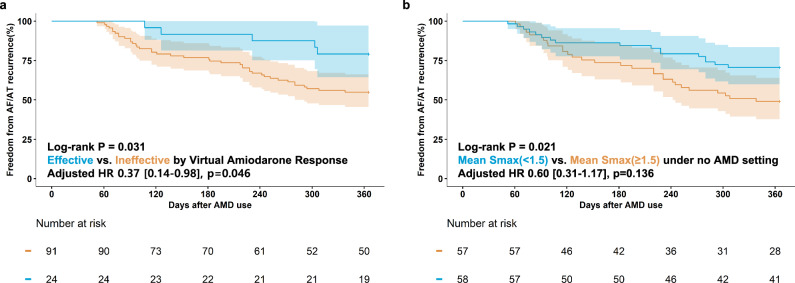
Kaplan–Meier analysis of AF recurrence after AMD use according to virtual AMD response and mean Smax values. **a** Graph divided by virtual AMD response (**b**) Baseline (No AMD): Graph divided by median value of mean Smax (1.5). AF atrial fibrillation, AMD amiodarone, Smax the maximal restitution slope of action potential duration.

To evaluate the clinical relevance of AMD using the digital twin, we categorized patients based on their response to therapeutic concentrations of the drug. In Fig. 2, cases where AF termination occurred at least once at either a low or high dose of AMD were classified as the virtually effective AMD group *(Effective)*. In contrast, cases where AF or AT persisted at therapeutic doses were classified as the virtually ineffective AMD group *(Ineffective)*. Supplementary Fig. 1 presents representative outcomes of virtual AF induction across varying AMD concentrations for both the *Effective* and the *Ineffective* groups. The percentage of cases classified into the *Effective* group was 20.9% (24/115), while the *Ineffective* group comprised 79.1% (91/115) of cases. Table 1 presents a comparison of the clinical and procedural characteristics of patients classified by virtual AMD response. No significant differences in baseline clinical or procedural characteristics were observed between the two groups. The Kaplan–Meier analysis revealed a significant difference in the 1-year clinical recurrence rates of AF/AT between the two groups (log-rank p = 0.031; Fig. 4a). The 1-year rhythm outcome was worse in patients with a mean Smax ≥1.5 than in those with a mean Smax <1.5 (log-rank p = 0.021, Fig. 4b). The multivariate analysis showed that the effective virtual AMD test was independently associated with lower clinical recurrence of AF/AT (adjusted hazard ratio 0.37, 95% confidence interval: 0.14-0.98; p = 0.046; Table 2). Harrell’s C-index was 0.700 [0.662-0.738], confirming the predictive value of virtual AMD digital twins for clinical rhythm outcomes. The final area under the receiver operating characteristic curve of the predictive model to determine the maintenance of sinus rhythm 1 year after AMD administration was 0.769 [0.679–0.858] (Supplementary Fig. 2, Model 3).

**Table 1:** Clinical and procedural characteristics of study population according to virtual AMD test

Variables	Overall	Effective^a^	Ineffective^b^	*P*-value
	*N* = 115	*N* = 24	*N* = 91	
Age, year	60.8 ± 10.0	62.6 ± 8.1	60.4 ± 10.4	0.324
Female, *N* (%)	31 (27.0)	7 (29.2)	24 (26.4)	0.987
Paroxysmal AF, *N* (%)	30 (26.1)	10 (41.7)	20 (22.0)	0.091
BMI, kg/m2	25.3 [23.2, 27.1]	25.9 [24.3, 27.7]	25.3 [23.2, 26.9]	0.399
Comorbidities				
CHF, *N* (%)	47 (40.9)	10 (41.7)	37 (40.7)	>0.999
HTN, *N* (%)	57 (49.6)	12 (50.0)	45 (49.5)	>0.999
DM, *N* (%)	20 (17.4)	4 (16.7)	16 (17.6)	>0.999
Stroke, *N* (%)	19 (16.5)	5 (20.8)	14 (15.4)	0.741
CHA2DS2VASc	2.1 ± 1.4	2.3 ± 1.4	2.1 ± 1.4	0.614
Beta-blocker (%)	52 (45.2)	15 (62.5)	37 (40.7)	0.093
Echocardiography				
LA dimension, mm	45.3 ± 6.1	44.1 ± 7.6	45.6 ± 5.6	0.310
LAVI, ml/m2	48.3 ± 15.3	44.9 ± 16.3	49.2 ± 15.0	0.224
LVEF, %	60.6 ± 9.7	61.5 ± 11.3	60.4 ± 9.3	0.645
E/Em	10.6 ± 4.7	10.0 ± 3.0	10.7 ± 5.1	0.532
Procedure time [IQR], min	129 [115, 148]	121 [110, 133]	131 [115, 149]	0.191
Repeat-ablation (%)	29 (25.2)	4 (16.7)	25 (27.5)	0.412
Ablation lesion				
PVI (%)	115 (100.0)	24 (100.0)	91 (100.0)	>0.999
Cavo-tricuspid isthmus (%)	113 (98.3)	24 (100.0)	89 (97.8)	>0.999
SVC – RA (%)	114 (99.1)	24 (100.0)	90 (98.9)	>0.999
Extra-PV LA ablation^c^	79 (68.7)	14 (58.3)	65 (71.4)	0.326
FU duration after AMD [IQR] day	1009 [777, 1263]	947 [757,1262]	1040 [806, 1281]	0.375
Days from AFCA to AMD [IQR], day	14 [13,20]	14 [12,36]	14 [13,19]	0.761
Early recurrence after AFCA (%)	91 (79.1)	18 (75.0)	73 (80.2)	0.781
Recur as AF (% early recur)	73 (80.2)	11 (61.1)	62 (84.9)	0.052
Recur after AMD within 1 year (%)	46 (40.0)	5 (20.8)	41 (45.1)	0.055
Recur as AF (% 1 yr recur)	34 (73.9)	4 (80.0)	30 (73.2)	>0.999
Procedure related Complications^d^ (%)	5 (4.3)	0 (0.0)	5 (5.5)	0.541

*AF* atrial fibrillation, *AFCA* atrial fibrillation catheter ablation, *AMD* amiodarone, *BMI* body mass index, *CFAE* complex fractionated atrial electrogram, *CHF* congestive heart failure, *DM* diabetic mellitus, *E/Em* The ratio of early transmitral flow velocity (E) to early mitral annular velocity (Em), *FU* follow-up, *HTN* hypertension, *IQR* Interquartile range, *LA* left atrium, *PV* pulmonary vein, *PVI* pulmonary vein isolation, *SVC* superior vena cava, *RA* right atrium.

^a^Effective group: AF termination at least once at the therapeutic dose.

^b^Ineffective group: AF or AT maintenance for >32 s at the therapeutic dose.

^c^Extra-PV LA ablation includes roof line, posterior-inferior line, left lateral isthmus, coronary sinus, anterior septal, anterior lateral, CFAE, or extra PV trigger ablation.

^d^Complications include 2 sinus node dysfunction, 2 pericardial tamponade, and 1 arteriovenous fistula.

**Table 2:** Cox regression analysis for atrial fibrillation/tachycardia recurrence within 1 year after AMD prescription

Variables	Univariable		Model 1 (*N* = 115)		Model 2 (*N* = 115)		Model 3 (*N* = 115)	
	HR, 95% (CI)	*P* value	HR, 95% (CI)	*P* value	HR, 95% (CI)	*P* value	HR, 95% (CI)	*P* value
Age, per SD increase	0.83 (0.63–1.10)	0.200	0.80 (0.58–1.11)	0.183	0.83 (0.60–1.15)	0.258	0.84 (0.61–1.17)	0.312
Female	1.06 (0.56–2.01)	0.868	1.04 (0.49–2.20)	0.922	1.00 (0.72–1.40)	0.986	1.02 (0.73–1.42)	0.919
Paroxysmal AF	1.08 (0.57–2.06)	0.804	0.79 (0.37–1.71)	0.553	0.87 (0.62–1.24)	0.443	0.91 (0.64–1.3)	0.618
BMI, per SD increase	1.13 (0.84–1.53)	0.421	1.12 (0.81–1.55)	0.497	1.01 (0.73–1.38)	0.974	1.06 (0.77–1.46)	0.719
Repeat ablation	1.84 (1.01–3.36)	0.046	2.02 (0.97–4.19)	0.060	1.45 (1.06–1.99)	0.020	1.45 (1.06–1.97)	0.020
Congestive heart failure	1.49 (0.84–2.66)	0.176	2.75 (1.32–5.74)	0.007	1.49 (1.04–2.15)	0.032	1.49 (1.04–2.14)	0.029
Hypertension	0.91 (0.51–1.63)	0.751	1.60 (0.77–3.33)	0.206	1.31 (0.91–1.88)	0.152	1.26 (0.88–1.8)	0.210
Diabetes mellitus	0.39 (0.14–1.08)	0.069	0.32 (0.11–0.96)	0.042	0.64 (0.42–0.99)	0.043	0.65 (0.42–0.99)	0.044
LA dimension, per SD increase	1.02 (0.77–1.36)	0.888	0.91 (0.62–1.35)	0.651	0.95 (0.64–1.40)	0.787	0.95 (0.65–1.4)	0.806
LVEF, per SD increase	1.22 (0.89–1.68)	0.215	1.59 (1.11–2.28)	0.012	1.56 (1.09–2.23)	0.016	1.51 (1.06–2.17)	0.024
Use of virtually effective AMD	0.37 (0.15–0.95)	0.038	0.37 (0.14–0.98)	0.046				
Mean Smax in no AMD, per SD increase	1.39 (1.05–1.84)	0.023			1.21 (0.87–1.70)	0.257		
Mean Smax in low AMD, per SD increase	1.32 (0.98–1.79)	0.072						
Mean Smax in high AMD, per SD increase	1.12 (0.83–1.51)	0.457						
Highest Mean Smax in no AMD, per SD increase	1.27 (0.94–1.71)	0.114						
Highest Mean Smax in Low AMD, per SD increase	1.05 (0.80–1.38)	0.707						
Highest Mean Smax in High AMD, per SD increase	0.92 (0.68–1.24)	0.579						
Lowest Mean Smax in no AMD, per SD increase	1.36 (1.07–1.73)	0.011					1.26 (0.96–1.64)	0.091
Lowest Mean Smax in Low AMD, per SD increase	1.27 (0.97–1.68)	0.086						
Lowest Mean Smax in High AMD, per SD increase	1.19 (0.91–1.56)	0.212						

*AF* atrial fibrillation, *AMD* amiodarone, *BMI* body mass index, *CI* confidence interval, *LA* left atrial, *LVEF* left ventricular ejection fraction, *SD* standard deviation.

### Regional differences of Smax and DF according to AMD dose and rhythm status

**Fig. 5:**
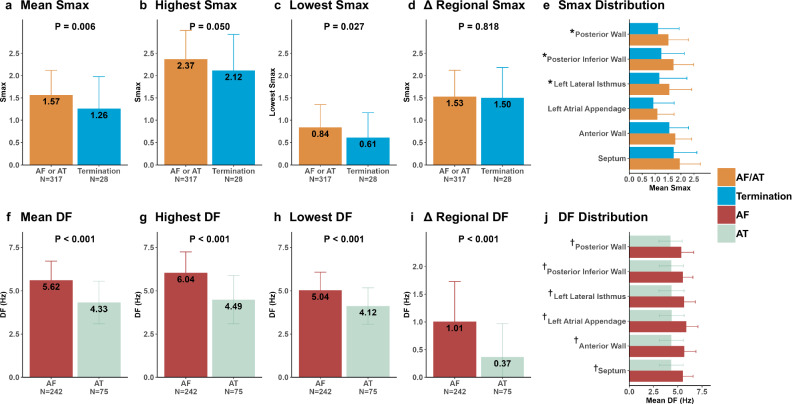
Mean, highest, lowest, and Δ regional Smax and DF according to LA region and virtual rhythm outcomes. All variables shown are expressed as mean and standard deviation. **a** Regional mean Smax and Mean Smax according to virtual rhythm outcomes. **b** The Highest Smax according to virtual rhythm outcomes. **c** The Lowest Smax according to virtual rhythm outcomes. **d** Δ Regional Smax according to virtual rhythm outcomes. **e** Mean Smax according to virtual rhythm outcomes and region. **f** Mean DF according to virtual rhythm outcomes. **g** The Highest DF according to virtual rhythm outcomes. **h** The Lowest DF according to virtual rhythm outcomes. **i** Δ Regional DF according to virtual rhythm outcomes. **j** Mean DF value according to virtual rhythm outcomes and region. *denotes that the p-value of difference between the two groups (AF/AT vs. Termination) is below 0.05. †denotes that the p-value of difference between the two groups (AF vs. AT) is below 0.05. AF atrial fibrillation, AT atrial tachycardia, DF dominant frequency, LA left atrial, Smax the maximal restitution slope of action potential duration.

Among 345 digital twin models (including no-, low-, and high-dose AMD), the mean Smax (1.57 ± 0.55 vs. 1.26 ± 0.71, p = 0.006), the highest regional Smax (2.37 ± 0.64 vs. 2.12 ± 0.80, p = 0.050), and the lowest regional Smax (0.84 ± 0.51 vs. 0.61 ± 0.56, p = 0.027) were significantly lower in the termination group than in the AF/AT sustaining group (Fig. 5a–c). However, Δregional Smax (mean Smax of the highest Smax region – mean Smax of the lowest Smax region) did not differ between the two groups (1.53 ± 0.59 vs. 1.50 ± 0.68, p = 0.818; Fig. 5d). In the regional analyses, regional Smax was significantly lower in the termination group than in AF/AT sustaining group at the left lateral isthmus (1.55 ± 0.87 vs. 1.16 ± 1.07, p-value = 0.027), posterior-inferior wall (1.71 ± 0.77 vs. 1.24 ± 0.89, p-value = 0.002), and posterior wall (1.52 ± 0.77 vs. 1.11 ± 0.82, p-value = 0.008) areas (Fig. 5e).

In the DF analyses, the mean DF, highest DF, lowest DF, and Δregional DF (mean DF of the highest DF region – mean DF of the lowest DF region) were consistently lower for AT cases than for AF (all p-values < 0.001; Fig. 5f–j).

ΔRegional Smax was reduced with virtual AMD (baseline 1.61 ± 0.53, low-dose AMD 1.54 ± 0.67, and AMD 1.43 ± 0.57, p for trend=0.024). The region with the highest Smax was consistently the septal area (31% at baseline, 37% at low-dose AMD, and 41% at high-dose AMD; Supplementary Fig. 3a), and that with the lowest Smax was the anterior wall (48.7% at baseline, 42.6% at low-dose AMD, and 41.7% at high-dose AMD; Supplementary Fig. 3b). In the DF analyses, the mean DF (p for trend <0.001), highest regional DF (p for trend <0.001), lowest regional DF (p for trend <0.001), and Δregional DF (p for trend <0.001) were significantly reduced in a dose-dependent manner (Supplementary Table 1).

## Discussions

In this study, we utilized the patient-specific AF digital twin, created from personal imaging and electrophysiological data, to test the effects of virtual AMD at various concentrations following ablation. Our findings showed that as the concentration of virtual AMD increased, the peak upstroke velocity of left atrial cells decreased, and the action potential duration increased, reflecting effects similar to those expected in clinical settings. Additionally, higher AMD concentrations were associated with decreases in surrogate markers for AF drivers, such as DF, and in Smax, which indicates wave-break properties. Furthermore, the virtual rhythm outcomes revealed that higher concentrations of virtual AMD led to more frequent AF termination and reduced AF maintenance. Finally, we observed significantly fewer 1-year clinical AF/AT recurrences in the group of patients for whom virtual AMD was effective in the digital twin study. The digital twin-guided virtual AMD test has a predictive value for the effectiveness of clinical AMD in AF rhythm control in patients undergoing AFCA.

In clinical electrophysiology, the integration of digital twin technology offers a transformative approach by creating virtual replicas of organs or individuals^9^, facilitating the simulation of personalized health scenarios. Zhang et al.^23^ employed late gadolinium enhancement (LGE)-MRI to create genotype-specific digital twins of patients with arrhythmogenic right ventricular cardiomyopathy, predicting ventricular tachycardia reentrant circuits with over 90% precision, which was validated using electrophysiology laboratory procedures. Other studies^11,24^ utilized patient-specific digital twins to predict AFCA outcomes and effectively ablate high-DF sites, highlighting the value of personalized models in cardiac intervention.

Previously, we utilized digital twins to delve into fundamental electrophysiological mechanisms^25^ to identify optimal ablation targets^14,15,17,26^ and predict responses to AADs^19,20^. However, a notable challenge in digital twin construction lies in the accurate modeling of fibrotic tissue^27^. While most approaches rely on assumptions derived from LGE-MRI data, issues such as thin atrial walls and limited resolution may compromise predictive accuracy^28^. We employed endocardial bipolar voltage measurements to delineate fibrotic states. Despite potential limitations associated with catheter positioning and electrode influences, we benefit from being able to closely replicate a patient’s actual conduction using clinical activation time and voltage maps. Notably, the absence of assessments of histological biopsies in both methods poses challenges for the precise evaluation of fibrosis. Nevertheless, ongoing efforts towards refining assessment techniques, such as new methods for optimizing image intensity thresholds^28^, hold promise for enhancing the concordance between observed low-voltage areas and image intensity ratios obtained by MRI.

Previous studies have demonstrated that AMD effectively flattens Smax restitution^29,30^ and prolongs APD without increasing dispersion^31^, thereby enhancing AF suppression. Our group has previously reported that a higher Smax (≥1.4) under AADs is associated with significantly lower DF^19^, and DF-guided ablation outcomes after PVI^17^ are superior when Smax is less than 1. In this study, using a therapeutic dose of AMD, we observed a dose-dependent decrease in Smax, with significant reductions at higher doses following virtual PVI. Notably, cases with AF termination exhibited a lower mean Smax compared to cases with sustained AF. Additionally, DF analysis during the 17–23 second interval revealed a consistent decrease in mean DF across all regions in cases with regular AT, supporting the efficacy of AMD in modifying substrate properties to prevent AF maintenance.

The limitations of using AAD lie in not only their moderate efficacy and side effects^32^ but also the inability to predict their efficacy prior to administration; leveraging a digital twin study allows for a more comprehensive understanding of AF mechanisms and facilitates comparison of AAD efficacy. The in silico model demonstrated favorable outcomes by showing that specific modifications^6^ or blockade^7^ of ionic currents reduced functional reentries. Computational modeling showed that AMD was more effective than dronedarone^7^. In one study, an integrated tool^33^ was developed to evaluate the effects of diverse AADs by modeling various species and diseases. Dasi et al.^34^ constructed a model of healthy human atria using electrocardiographic data to assess the efficacy of AMD. By performing a digital twin study with the CUVIA (Clinical Usefulness of Virtual Ablation Guided Catheter Ablation of Atrial Fibrillation, Laonmed, Inc., Seoul, South Korea) program, we previously reported that efficacies and anti-AF effects of diverse AADs could be assessed depending on genetic traits^18–20^. To our knowledge, this is the first retrospective study to assess the efficacy of AMD and match the virtual test results with actual prescribed AMD in a clinical setting. We compared rhythm outcomes in patients with virtual AMD test results 3–12 months post-AFCA. We observed that the use of virtually effective AMD was an independent predictor of AF recurrence within 1 year of follow-up after AMD treatment.

In digital twin technology, the importance lies in the accuracy of the model and reproducibility of the results. First, to enhance patient-specific accuracy, it is essential to improve current-voltage mapping methods. Utilizing techniques such as charge density mapping^35^ or machine learning algorithms during voltage mapping to differentiate fibrotic tissue^36^ can significantly enhance accuracy. However, since invasive procedures cannot be performed on all patients who do not need ablation, integrating non-invasive data, including that from 12-lead electrocardiography^37^, with artificial intelligence (AI) technology to augment mapping data accuracy to at least the current level would diversify digital twin studies.

Second, reducing the time required for simulations is crucial for demonstrating reproducibility. Currently, simulations are primarily conducted by skilled researchers and require considerable manual effort and time. The growth of digital twin technology is poised to skyrocket, with the potential to automate all processes, including experimenting with diverse concentrations^38^, adjusting pacing locations^12^, modifying pacing protocols^39^, and entrusting AI to determine the AF maintenance status^40^. This automation would not only enhance versatility and reproducibility but also drive significant advancements in the field.

However, we evaluated the efficacy of only one AAD—AMD—in this study. We aimed to evaluate the electrophysiological changes and effects of utilizing different AADs in digital twin simulations. If automated through the AI technology mentioned earlier and constructed via noninvasive methods, digital twin models could potentially be applicable to general patients without the requirement to undergo procedures. Therefore, the predictive capability of the virtual AMD test needs to be validated in prospective randomized studies.

Our study has several limitations. First, this was a single-center, retrospective, observational study. Although the virtual AMD test results independently predicted the 1-year rhythm outcome, there may have been an inherent selection bias due to our inclusion of patients with a high likelihood of AF recurrence who were prescribed AMD. Second, our digital twin implemented only the LA in a monolayer. Our previous work^41^ using a bi-atrial model, which included the right atrium, highlighted the critical role of interatrial conductions in AF maintenance. The lack of imaging techniques with sufficient resolution to accurately assess interatrial conduction, combined with the routine use of LA mapping in clinical practice, led us to develop an LA-specific digital twin. Third, as previously mentioned, fiber orientation was implemented based on the atlas, with conduction velocity manually adjusted according to the propagation direction. To evaluate the inter-observer variability of virtual rhythm outcomes based on fiber orientation and LAT synchronization, we calculated Fleiss’ Kappa coefficient, which was 1 among three observers across 10 cases, indicating very little variation. Fourth, we did not perform virtual ablation tailored to the specific ablation lesions of each patient, which is another limitation of this study. Subgroup analysis of the virtual AMD response relative to ablation lesions revealed p-values for interaction all above 0.05, indicating no statistical significance (Supplementary Table 2). Fifth, the proportion of patients with AF termination is low, but consistent to the previous reports^34^. While AF termination with virtual AMD reflects the drug’s potency in effectively blocking AF maintenance mechanisms, there is no direct evidence linking these findings to long-term clinical outcomes.

In conclusion, using the digital twin-guided virtual AMD test, we could predict the AMD responses before its administration. Furthermore, based on the results of the virtual AMD test, we observed a reduction in the 1-year recurrence rate by prescribing virtually effective AMD for each patient. Additionally, to address the limitations of this retrospective study, a multicenter prospective randomization study is underway to assess the predictive power of the digital twin virtual AMD test.

## Methods

### Study population

This study was approved by the Institutional Review Board of the Severance Cardiovascular Hospital, Yonsei University Health System, and conducted in accordance with the principles of the Declaration of Helsinki. All patients in the Yonsei AF Ablation Cohort Database (ClinicalTrials.gov Identifier: NCT02138695) provided written informed consent for their clinical data to be used in digital twin studies. We followed the Strengthening the Reporting of Observational Studies in Epidemiology (STROBE) reporting guideline^42^.

To select patients with AF for whom CT and EAM could be performed, this study was conducted on those who underwent AMD treatment for recurrent or symptomatic non-sustained AT within 3 months after AFCA. Between April 2014 and October 2021, 681 patients who underwent AFCA and were eligible for a digital twin study were initially enrolled. The final cohort comprised individuals who underwent AMD treatment within 3 months and were followed up for up to 1 year from the commencement of AMD therapy. Individuals who abstained from AAD treatment post-AFCA, patients for whom AAD was initiated more than 3 months after ablation, patients treated with drugs other than AMD, patients with a follow-up duration <1 year, and cases without additional analysis in the digital twin study were excluded. Finally, 115 patients were included in the analyses (Fig. 2).

### Clinical substrate mapping and AF ablation

At AFCA initiation, we created LA substrate maps (comprising bipolar voltage and local activation maps) using the EnSite NavX system, obtained with a multielectrode catheter (AFocus, Abbott, Chicago, IL, USA) during high right atrial (RA) pacing, with a cycle length of 500 ms. If AF persisted, we performed internal cardioversion using biphasic shock (2–20 J) with R-wave synchronization (Lifepak12, Physiocontrol Ltd., Redmond, WA, USA) to restore sinus rhythm. Cases in which sinus rhythm could not be restored, PVI was performed, followed by mapping after sinus rhythm restoration. We collected bipolar electrogram data from the LA surface and obtained more than 500 points per patient.

An open-irrigated tip catheter (FlexAbility, Abbott Inc.; Coolflex, Abbott Inc., Minnetonka, MN, USA; 30–35 W; 47 °C; TactiCath, Abbott Inc.; and ThermoCool SmartTouch, Biosense Webster Inc.) was employed for AFCA. The ablation endpoint at each site was defined as an average impedance drop >10% of the baseline or a >80% decrease in the local electrogram voltage amplitude. We achieved circumferential PVI (CPVI) using a bidirectional block in all patients. Additional lesion formation beyond CPVI was determined at the operator’s discretion.

### Follow-up strategy and AMD prescription

After the procedure, all patients were monitored regularly following the same schedule as that followed for previous patients who underwent AF ablation in our center^43^. AMD was prescribed, if no contraindications were present, to patients with early recurrence of AF or AT within 3 months, symptoms of suspected arrhythmia, or ineffective cardioversion during the procedure. Typically, AMD was administered at a dose of 200 mg once daily for 2–6 weeks, followed by a reduction to 100 mg if rhythm control was achieved, which was then maintained. Patients who underwent post-AFCA AMD treatment were followed up at 1, 3, and 6 months after the procedure and every 6 months thereafter.

### Creating a digital twin of the LA

We created a digital twin of the LA by merging pre-ablation CT images with EAM data obtained during AFCA.

### 3D Geometry formation

The structural components of the geometry were represented as triangular meshes, with each node forming a human atrial myocyte (Fig. 1b). First, triangular meshes were developed based on individual LA CT images. These 3D mesh surfaces consisted of ~400,000–500,000 nodes, with a mean spacing of 235.1 ± 32.1 μm between adjacent nodes. Throughout the AFCA procedure, we collected bipolar electrogram data from >500 points on the LA surface using a circular mapping catheter and an Ensite NavX system (Abbott Inc., Chicago, IL, USA), during a paced rhythm with a cycle length of 500 ms. We aligned the coordinates of the EAM with individual CT images, producing a clinical EAM. Additional details regarding the procedure with aligning EAM with CT images were described in the previous study^44^.

### Human atrial myocyte model and action potential propagation

We used a modified Courtemanche-Ramirez-Nattel (mCRN) model^45,46^ to characterize the system, which mathematically represents the various ion currents within human atrial myocytes, as shown in Eq. (1).

1 $$\begin{array}{l}{I}_{{ion}}={I}_{{Na}}+{I}_{{CaL}}+{I}_{{to}}+{I}_{{Kur}}+{I}_{{Kr}}+{I}_{{Ks}}+{I}_{K1}+{I}_{{K}_{{Ach}}}\\\qquad\;\; +\,{I}_{{NaCa}}+{I}_{{NaK}}+{I}_{b,{Na}}+{I}_{b,{Ca}}+{I}_{p,{Ca}}\end{array}$$

Where I_ion_ denotes the ionic current of the atrial myocyte, comprised of various individual ion channel currents—including sodium (I_Na_), potassium (I_to_, I_Kur_, I_Kr_, I_Ks_, I_K1_, I_KAch_), calcium (I_CaL_), exchanger (I_NaCa_), pump (I_NaK_, I_p,Ca_), and background currents (I_b,Na_, I_b,Ca_). This model served as the foundation for generating action potentials and simulating wave propagation in atrial myocytes.

Assuming each cardiac cell represents a single node, a triangular array was created with nodes representing cell-to-cell connections. To computationally model cardiac action potential propagation through the atrial wall, we used the following reaction-diffusion equation^47^ referred to as Eq. (2).

2 $$\frac{\partial {V}_{m}}{\partial {\rm{t}}}=\nabla \,\cdot\, D\nabla {V}_{m}-\frac{{I}_{{ion}}+{I}_{{stim}}}{{C}_{m}}$$

Where V_m_ (V) denotes the membrane potential, C_m_ (F/m²) represents the membrane capacitance per unit area, D (m^2^/s) is the diffusion coefficient, and I_stim_ (ampere/meter²) refers to the stimulation current. To simulate the reaction-diffusion system in 3D, we used models constructed along a generalized finite difference scheme.

### Electrophysiological and histological characterization

After geometry formation, the electrophysiological characterization of each node involved determining parameters, such as voltage, fibrosis state, fiber orientation, and conductivity (Fig. 1b). Voltage interpolation was performed for clinical voltage data using the inverse distance weighting method as shown in Eq. (3)^48^

3 $${W}_{{ij}}=\frac{{{d}_{{ij}}}^{-a}}{{\sum }_{k}^{{n}_{j}}{d}_{{kj}}},{R}_{j}=\mathop{\sum }\limits_{i=1}^{{n}_{j}}{w}_{{ij}}{R}_{{ij}}$$

Where W represents the weighted average of neighboring values, i and j indicate the known and unknown values of the points, *dij*^− a^ is the distance between known and unknown points, *Rij* represents the value of the known point, and *Rj* indicates the interpolated value at the unknown point j. The interpolation process produced the virtual voltage data with an amplitude within a 10-mm radius from the region of interest.

The fibrotic state was determined by applying the obtained virtual voltage values to a probability function, to distinguish fibrotic cells from normal cells. To determine fibrosis status (yes/no) for each node, we used the following nonlinear equation (Eq. (4)) between the bipolar voltage and probability of fibrosis:

4 $${P}_{{fibrosis}}=\left\{\begin{array}{cc}1 & V\, <\, 0\\ \frac{1}{100}* (-40{V}^{3}+155{V}^{2}-206V+99.8) & 0\le V\le 1.74\\ 0 & 1.74\, <\, X\end{array}\right.$$

where *P*_*fibrosis*_ is the probability that there is fibrosis at a given node, and virtual *V* is the bipolar voltage at that node within the 0–1.74 mV range. If *V* is >1.74 mV, *P*_*fibrosis*_ converges to zero. This was developed by comparing the predicted percentage of fibrosis across the 3D atrial model with pre- and post-ablation fibrosis data. For each node, the probability of fibrosis calculated based on the clinically acquired bipolar voltage data was compared against a random number between 0–1. In fibrotic cells, ion currents—including the inward rectifier potassium current (I_K1_), L-type calcium current (I_CaL_), and sodium current (I_Na_)—were reduced by 50%, 50%, and 40%, respectively^12^.

For fiber orientation, we used an atlas^49^ based on the fiber orientation of the LA surface and compared it with the CUVIA program to adjust for patient-specific geometries. Representative vectors were drawn, and fiber orientations at surrounding nodes were determined through interpolation around these representative vectors. A vector aligned with the myocardial fiber direction was generated at each point in the heart. Conductivity was set based on orientation, differentiation between the longitudinal and transverse directions, and fibrotic and non-fibrotic tissues^12^. We defined the longitudinal conduction velocity as that in the same direction as the vector and the transversal conduction velocity as that in the perpendicular direction to the vector. The conductivity of the model was applied at 0.1264 S/m (non-fibrotic longitudinal cell), 0.0546 S/m (fibrotic longitudinal cell), 0.0252 S/m (non-fibrotic transverse cell), and 0.0068 S/m (fibrotic transverse cell).

Finally, to synchronize the clinical and virtual LAT maps and determine the conduction velocity, we displayed the clinical LAT map on the CUVIA program screen. An experienced investigator synchronized the virtual LAT map to match its appearance to that of the clinical map. To assess inter-observer variability, we compared LATs synchronized by two different investigators across 10 cases and fiber orientation maps created by three different investigators. After inducing virtual AF, we compared the outcomes. We found that all 10 cases resulted in the same final rhythm (AF, AT, or termination), regardless of the synchronization or fiber orientation method used. Fleiss’ Kappa coefficient for inter-observer variability was calculated to be 1, indicating perfect agreement. By adjusting the diffusion coefficients of the 3D model to match the conduction velocity of the virtual LAT map with that of the clinical LAT map, we were able to induce virtual AF and interventions in the digital twin.

### Virtual PVI and AMD intervention

Using the CUVIA digital twin, circular lesions of 2 mm width were created on both sides of the PVs (Fig. 1c). We conducted PVI at the antral levels. In the presence of AF, which is characterized by distinct ion current adaptations compared with sinus rhythm, adjustments were made to the conductance or concentration of specific ion channels (Supplementary Table 3). Considering the increased recurrence risk (early recurrence, symptoms, or challenges in achieving sinus rhythm during the procedure) among enrolled patients, we tailored the diffusion coefficient of the control model to ensure the persistence and maintenance of AF in all simulations during the observation period (32 seconds).

To conceptualize the conditions under which AMD acts within the body at subtoxic ranges, we defined low, high, and toxic doses of AMD as 1.6 μM (minimal effective concentration), 3.9 μM (maximal effective concentration), and 8.0 μM (toxic concentration) respectively (Fig. 1d), based on the therapeutic range of AMD^50^. Given the role of AMD as a multiple ion channel blocker, we investigated the functional blockade of ion channel conductance at these concentrations. The degree of functional blockade was evaluated using Hill’s equation^8,33^, expressed as Eq. (5).

5 $$\theta ={[1+{({{IC}}_{50}/D)}^{{nH}}]}^{-1}$$

Where *θ* denotes the degree of channel blockade (ranging from 0 to 1), *IC*_*50*_ represents the half-maximal inhibitory concentration, *D* is the free drug concentration, and *nH* is the Hill coefficient. The ion channel specific IC_50_, Hill’s coefficient, and corresponding references, along with the ion current settings for low (1.6 μM), high (3.9 μM), and toxic (8.0 μM) AMD concentrations, are summarized in Supplementary Table 3.

### Evaluation of electrophysiologic parameters depending on virtual AMD

To assess electrophysiological changes under AMD treatment, we conducted pacing at the earliest activation site (EAS) with a cycle length of 500 ms (Fig. 1e). We measured APD_90_ and peak upstroke velocity across baseline, low-, high-, and toxic-dose AMD scenarios near the activation site. Subsequently, we compared the mean values and assessed whether a dose-dependent trend was observed.

Next, to assess the effectiveness of AMD in patients, all conditions (baseline, low-, high-, and toxic-dose AMD) were subjected to ramp pacing to induce AF (Fig. 1g). To induce virtual AF, we ramped pacing around the EAS for 11.52 s with 8 beats/cycle. The pacing started at 200 ms and was decreased at 10 ms intervals until it reached 120 ms.

We created the Smax and DF maps for the aforementioned scenarios as shown in Fig. 1g. To determine the Smax, the APD_90_ and DI were measured at each node during a pacing period of up to 3 beats after a wave break during rapid pacing (200 ~ 120 ms)^26^. Smax was calculated as the maximum slope of the APD_90_ restitution curve and defined for all nodes (>400,000) in the LA model. The nonlinear fitting of the APD_90_ and DI was calculated using the following correlation equation, Eqs. (6) and (7).

6 $$y\left({Action\; potential\; duation}\right)={y}_{0}+{A}_{1}\left(1-{e}^{-\frac{{DI}}{{\tau }_{1}}}\right)$$

7 $${slope}=\left(\frac{{A}_{1}}{{\tau }_{1}}\right)* {Exp}\left(-\frac{{DI}}{{\tau }_{1}}\right)$$

Where *y*_*0*_ and *A*_*1*_ are free-fitting variables, and *τ*_*1*_ is a time constant. In each patient, we obtained the Smax value of each node.

The DF was derived from a Fourier transform of the action potentials for 6 s (17–23 s after the initiation of ramp pacing) at each node^15^. We evaluated the DFs for all nodes of the LA model.

After virtual PVI, the remaining LA was divided into 6 regions (septum, anterior wall, left atrial appendage, left lateral isthmus, posterior wall, posterior inferior wall) per patient to perform regional analysis (Fig. 1f)^25^. In each region, we analyzed the average Smax and DF values of each regions (Fig. 1g). We also analyzed the highest and lowest mean Smax and DF values among the six regions and compared regional differences by calculating Δregional Smax (mean Smax of the highest Smax region – mean Smax of the lowest Smax region) according to concentration or final rhythm (Fig. 5, Supplementary Table 1, Supplementary Fig. 3).

### Correlating the effects of virtual and clinical AMD

We observed whether the virtual AF persisted for 32 s, including the pacing time. Termination was defined as the absence of an activation signal at the final observation time (32 s). Conversion to AT was defined as the change from AF to regular tachycardia during the observation period (Fig. 1f). Based on the virtual AMD test results, individuals were categorized into the *Effective group* if AF termination occurred at least once at therapeutic concentrations; otherwise, they were classified into the *Ineffective group* (Supplementary Fig. 1). Subsequently, we conducted a comparative analysis of the virtual *Effective* and *Ineffective* groups to assess the clinical recurrence of AF or AT from the actual date of AMD prescription until 1 year later. A model was created to predict the probability of maintaining sinus rhythm 1 year after AMD treatment.

### Statistical analysis

Continuous variables without a normal distribution are presented as medians and interquartile ranges, and variables with a normal distribution are presented as means ± standard deviations. Continuous variables without a normal distribution were analyzed using the Mann-Whitney U test for two-group comparisons and the Kruskal-Wallis test for three or more group comparisons. Continuous variables with a normal distribution were examined using a t-test for two-group comparisons and analysis of variance to compare three groups.

We conducted paired t-tests or Wilcoxon rank-sum tests to assess changes in continuous variables between the virtual AMD test groups. Additionally, we used the Cochran-Armitage test for trend analysis and a linear regression analysis for examining differences in responses based on AMD concentration. A Kaplan–Meier analysis with a log-rank test was used to analyze AF recurrence-free survival over time and compare recurrence rates among the groups. To identify predictors associated with clinical AF recurrence after 1 year of AAD use, a multivariate Cox regression analysis was performed. To assess the predictive ability of maintaining sinus rhythm 1 year after the virtual AMD test, we calculated the area under the receiver operating characteristic curve. Statistical significance was set at a two-sided P-value < 0.05. All statistical analyses were performed using R version 4.2.3 (R Foundation for Statistical Computing, Boston, Massachusetts, United States).

## Supplementary information

Supplementary information

## Data Availability

The data collected and analyzed in this study are only available from the corresponding author upon reasonable request and with permission of the institution review board.

## Code availability

All simulations were conducted utilizing our local CUVIA program. Statistical analyses were carried out using R (v4.2.3), employing default codes and packages commonly utilized within the R environment. Access to the statistical analysis codes utilized is available from the corresponding author upon reasonable request.
