# Supplementary material for: Clinical usefulness of digital twin guided virtual amiodarone test in patients with atrial fibrillation ablation
Source: NPJ Digit Med. 2024 Oct 23;7:297. doi: 10.1038/s41746-024-01298-z (PMC11499921; doi:10.1038/s41746-024-01298-z)
Supplement: Supplementary file 1 — Supplementary information [file 41746_2024_1298_MOESM1_ESM.pdf]

Supplementary Information.

This material has been provided by the authors to give readers additional information about their work.

Table of contents

Supplementary Figure 1. Representative virtual outcomes of amiodarone in *Effective* and *Ineffective* groups

Supplementary Figure 2. ROC curve for 1-yr maintenance of sinus rhythm

Supplementary Figure 3. Proportions of the highest and lowest Smax region according to the amiodarone concentration and virtual response

Supplementary Table 1. Electrophysiologic parameters and rhythm outcomes according to amiodarone concentration

Supplementary Table 2. LA ablation lesion sets and subgroup analysis

Supplementary Table 3. The maximal conductance for various ion currents during sinus rhythm, AF with and without low or high amiodarone condition with IC50 and Hill's coefficient

Supplementary References

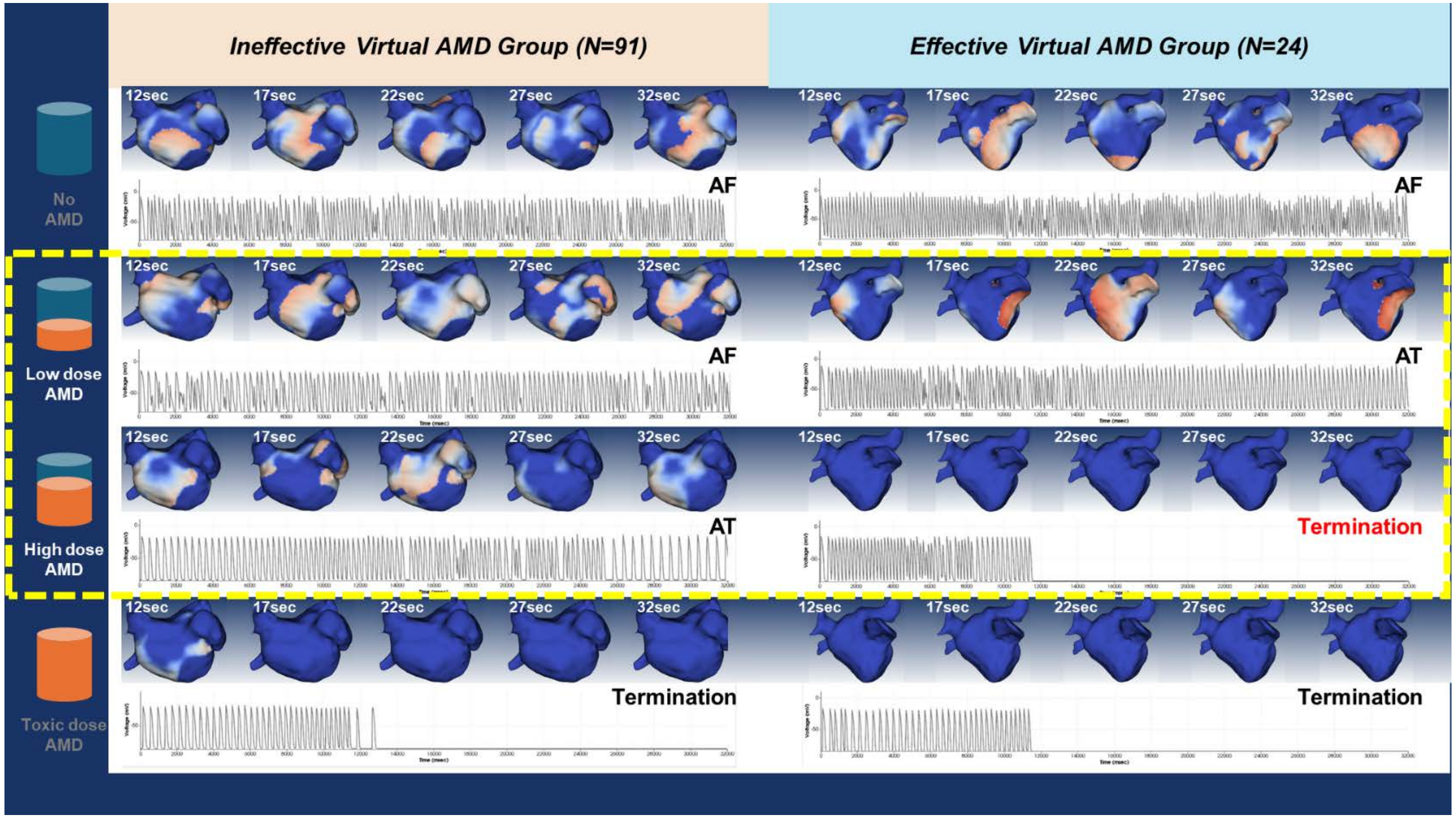

Supplementary Figure 1. Representative virtual outcomes of amiodarone in *Effective* and *Ineffective* groups

This figure illustrates the virtual outcomes of amiodarone (AMD) at various concentrations in the ***Ineffective*** Virtual AMD Group (N=91) and the ***Effective*** Virtual AMD Group (N=24). Both groups demonstrated AF maintenance in the absence of AMD and termination at toxic doses. Notably, the ***Effective*** group showed AF termination at high AMD doses, whereas the ***Ineffective*** group did not exhibit termination at either low or high doses.

AF, atrial fibrillation; AMD, amiodarone; AT, atrial tachycardia; APD, action potential duration

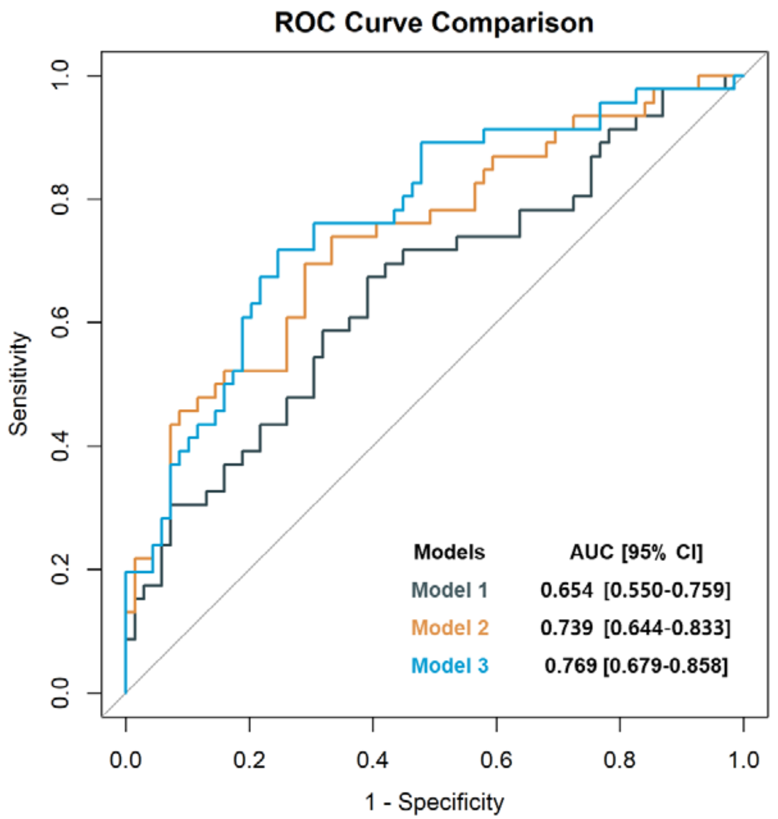

Supplementary Figure 2. Receiver operating characteristic (ROC) curve for 1-yr maintenance of sinus rhythm
Prediction Model 1 utilized variables including age, sex, body mass index, paroxysmal AF, repeat ablation, LA dimension, and left ventricular ejection fraction.
Prediction Model 2 encompassed all variables used in Model 1, with the addition of a history of congestive heart failure and diabetes.
Prediction Model 3 extended Model 2 by also including virtual AMD effectiveness.
AF, atrial fibrillation; AMD, amiodarone; AUC, area under curve; LA, left atrial

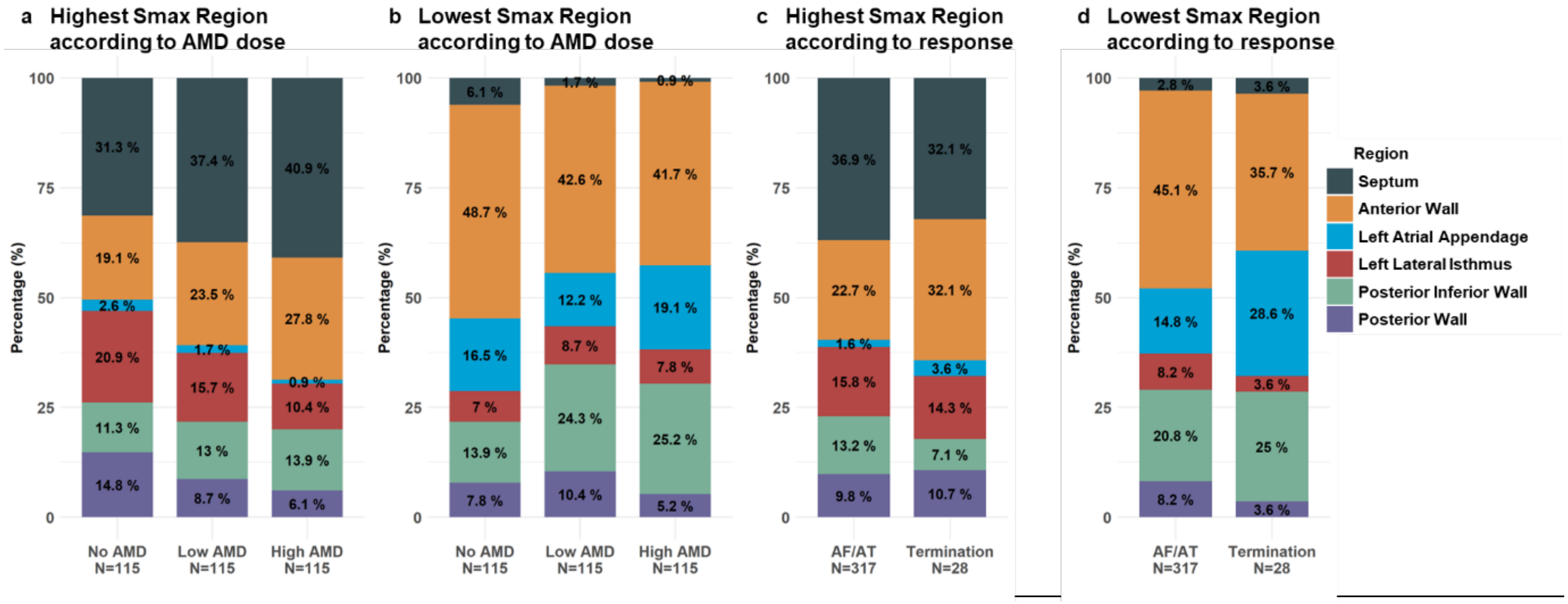

Supplementary Figure 3. Proportions of the highest and lowest Smax regions according to the amiodarone concentrations and virtual responses

(a) The proportions of the highest Smax region according to AMD dose. (b) The proportions of the lowest Smax region according to AMD dose. (c) The proportions of the lowest Smax region according to virtual AMD response. (d) The proportions of the lowest Smax region according to virtual AMD response

Supplementary Table 1.-Electrophysiologic parameters and rhythm outcomes according to amiodarone concentration

	No AMD (0 μM)) N=115	Low AMD (1.6 μM) N=115	High AMD (3.9 μM) N=115	P-value for Low vs. High	P-value for ANOVA	P for trend
Pacing condition: Pacing at Bachman's bundle with 500ms Cycle length						
APD_{90} (msec)	173.9±26.7	195.7±31.0*	200.8±34.6*	0.233	<0.001	<0.001
dV/dt_{max} (V/sec)	31.5±6.0	26.2±5.4*	21.9±4.9*	<0.001	<0.001	<0.001
Pacing condition: Ramp pacing at Bachman's bundle to induce AF						
Mean Smax	1.53±0.53	1.71±0.56*	1.38±0.58*	<0.001	<0.001	0.020
Septum	1.83±0.78	2.13±0.84	1.84±0.75	0.006	0.005	0.829
Anterior wall	1.64±0.65	1.96±0.56	1.68±0.67	0.001	<0.001	0.958
Left atrial appendage	1.05±0.64	1.22±0.67	0.96±0.63	0.003	0.008	0.205
Left lateral isthmus	1.65±0.92	1.67±0.86	1.24±0.84	<0.001	<0.001	<0.001
Posterior inferior wall	1.70±0.75	1.86±0.80	1.46±0.79	<0.001	0.001	0.010
Posterior wall	1.59±0.80	1.62±0.78	1.24±0.70	<0.001	<0.001	<0.001
Highest regional Smax	2.40±0.59	2.51±0.67	2.14±0.66*	<0.001	<0.001	<0.001
Lowest regional Smax	0.79±0.48	0.97±0.55*	0.70±0.49	<0.001	<0.001	0.105
[†]Δ Regional Smax	1.61±0.53	1.54±0.67	1.43±0.57*	0.191	0.078	0.024
Eligible DF analysis	N=115	N=104	N=98			
Mean DF, Hz	6.59±1.23	5.33±1.30*	4.94±1.27*	0.034	<0.001	<0.001
Septum	6.53±1.23	5.27±1.25*	4.89±1.26*	0.033	<0.001	<0.001
Anterior wall	6.66±1.32	5.38±1.38*	4.96±1.34*	0.028	<0.001	<0.001
Left atrial appendage	6.86±1.34	5.51±1.45*	5.12±1.38*	0.055	<0.001	<0.001
Left lateral isthmus	6.47±1.27	5.36±1.38*	4.99±1.31*	0.054	<0.001	<0.001
Posterior inferior wall	6.48±1.26	5.26±1.27*	4.88±1.23*	0.030	<0.001	<0.001
Posterior wall	6.32±1.41	5.14±1.37*	4.76±1.34*	0.051	<0.001	<0.001
Highest regional DF	7.09±1.36	5.68±1.50*	5.24±1.42*	0.031	<0.001	<0.001
Lowest regional DF	5.93±1.12	4.86±1.12*	4.53±1.13*	0.037	<0.001	<0.001
[‡]Δ Regional DF	1.16±0.88	0.82±0.79*	0.71±0.70*	0.282	<0.001	<0.001
Virtual Rhythm outcomes						
Final rhythm (%) at 32 sec				0.473	<0.001	
AF	115 (100)	66(57.4)	61(53.0)			
AT	0 (0)	38(33.0)	37(32.2)			
Termination	0 (0)	11(9.6)	17(14.8)			
Termination Rate (%)	-	11(9.6)	17(14.8)	0.313		
AT Conversion Rate (%)	-	38 (33.0)	37(32.2)	>0.999		

APD_{90}, 90% of action potential duration; dV/dt_{max}, peak upstroke velocity, AF, atrial fibrillation; AMD, amiodarone; AT, atrial tachycardia; Smax, the maximal restitution slope of action potential duration

*: The difference between selected value and the value when there is No AMD is statistically significant at $p < 0.05$ (based on the result of the paired t-test or Wilcoxon signed-rank test)

†: Δ Regional Smax is calculated the mean Smax of the highest Smax region – mean Smax of the lowest Smax region.

‡: Δ Regional DF is calculated the mean DF of the highest DF region – mean DF of the lowest DF region.

Supplementary Table 2. LA ablation lesion sets and subgroup analysis

Variables	Overall N=115	Effective N=24	Ineffective N=91	P-value	P for interaction
Ablation lesion					
PVI (%)	115 (100.0)	24 (100.0)	91 (100.0)	>0.999	-
Posterior box lesion (%)	44 (38.3)	5 (20.8)	39 (42.9)	0.082	0.542
Anterior line (%)	26 (22.6)	4 (16.7)	22 (24.2)	0.611	0.380
Extra-PV LA ablation*	79 (68.7)	14 (58.3)	65 (71.4)	0.326	0.931

LA, left atrium; PV, pulmonary vein; PVI, pulmonary vein isolation

Supplementary Table 3. The maximal conductance for various ion currents during sinus rhythm, AF with and without low or high amiodarone condition with IC50 and Hill's coefficient

Ion currents or maximal conductance	SR (%*)	No AMD (%*)	IC50(μM)	nH	AMD 1.6 μM (%*)	AMD 3.9 μM (%*)	AMD 8.0 μM (%*)
Inward current							
G_{Na}	100	90[4]	4.84[5]	0.76	63	49	37
G_{CaL}	100	30[6]	5.8[7]	1	24	18	13
G_{K1}	100	210[8]			210	210	210
Outward current							
G_{to}	100	30[9]	3.8[10]	1	21	15	10
G_{Kur}	100	50[11]			50	50	50
G_{Kr}	100	100	2.8[12]	0.91	63	43	28
G_{Ks}	100	100	3.84[13]	0.63	63	50	39
Pump, exchanger, or sarcoplasmic reticulum related current							
I_{KAch}	100	100			100	100	100
$I_{NaK(max)}$	100	100	15.6[14]	1	91	80	66
$I_{NaCa(max)}$	100	100	3.3[15]	1	67	46	29
$Ca^{\dagger}_{up(max)}$	100	80[9]			80	80	80
$I_{up(max)}$	100	100			100	100	100
$I_{rel(max)}$	100	100			100	100	100

AF, atrial fibrillation; G_{Na}, maximal I_{Na} conductance; I_{Na}, Fast inward sodium current G_{CaL}, maximal I_{CaL} conductance, I_{CaL}, L-type inward calcium current; G_{K1}, maximal I_{K1} conductance; I_{K1}, Inward rectifier potassium current; G_{to}, maximal I_{to} conductance; I_{to}, Transient outward potassium current; G_{Kur}, maximal I_{Kur} conductance; I_{Kur}, Ultrarapid delayed rectifier potassium current; G_{Kr}, maximal I_{Kr} conductance; I_{Kr}, Rapid delayed rectifier potassium current; GKs, maximal I_{Ks} conductance; I_{Ks}, Slow delayed rectifier potassium current; I_{KAch}, Acetylcholine activated potassium current; I_{NaK}, maximal I_{NaK}; I_{NaK}, sodium-potassium pump current; $I_{NaCa(max)}$, I_{NaCa} scaling factor; I_{NaCa}, sodium-potassium exchanger current; $C_{aup(max)}$, maximal calcium concentration in uptake compartment of sarcoplasmic reticulum; I_{up}, calcium uptake current into the uptake compartment of sarcoplasmic reticulum; $I_{rel(max)}$, maximal calcium release current from the release compartment of sarcoplasmic reticulum; IC_{50} , Half-maximal inhibitory concentration; nH, Hill's coefficient; SR, sinus rhythm

Values are presented as numbers (percentages).

*, Maximal conductance of current of each ion channel.

†, calcium concentration

Supplementary References

1. Courtemanche, M., Ramirez, R.J. & Nattel, S. Ionic mechanisms underlying human atrial action potential properties: insights from a mathematical model. *The American journal of physiology* **275**, H301-321 (1998).
2. Kneller, J., *et al.* Cholinergic atrial fibrillation in a computer model of a two-dimensional sheet of canine atrial cells with realistic ionic properties. *Circulation research* **90**, E73-87 (2002).
3. Zozor, S., *et al.* A numerical scheme for modeling wavefront propagation on a monolayer of arbitrary geometry. *IEEE transactions on bio-medical engineering* **50**, 412-420 (2003).
4. Sossalla, S., *et al.* Altered Na(+) currents in atrial fibrillation effects of ranolazine on arrhythmias and contractility in human atrial myocardium. *J Am Coll Cardiol* **55**, 2330-2342 (2010).
5. Lalevée, N., Nargeot, J., Barrére-Lemaire, S., Gautier, P. & Richard, S. Effects of amiodarone and dronedarone on voltage-dependent sodium current in human cardiomyocytes. *J Cardiovasc Electrophysiol* **14**, 885-890 (2003).
6. Pandit, S.V., *et al.* Ionic determinants of functional reentry in a 2-D model of human atrial cells during simulated chronic atrial fibrillation. *Biophys J* **88**, 3806-3821 (2005).
7. Nishimura, M., Follmer, C. & Singer, D. Amiodarone blocks calcium current in single guinea pig ventricular myocytes. *Journal of Pharmacology and Experimental Therapeutics* **251**, 650-659 (1989).
8. Wilhelms, M., *et al.* Benchmarking electrophysiological models of human atrial myocytes. *Front Physiol* **3**, 487 (2012).
9. Grandi, E., *et al.* Human atrial action potential and Ca2+ model: sinus rhythm and chronic atrial fibrillation. *Circulation research* **109**, 1055-1066 (2011).
10. Crumb, W.J., Jr., Vicente, J., Johannesen, L. & Strauss, D.G. An evaluation of 30 clinical drugs against the comprehensive in vitro proarrhythmia assay (CiPA) proposed ion channel panel. *Journal of pharmacological and toxicological methods* **81**, 251-262 (2016).
11. Kim, Y.G., *et al.* Association of Antiarrhythmic Drug Therapy With Syncope and Pacemaker Implantation in Patients With Atrial Fibrillation. *J Am Coll Cardiol* **83**, 1027-

1038 (2024).

12. Kamiya, K., *et al.* Short-and long-term effects of amiodarone on the two components of cardiac delayed rectifier K+ current. *Circulation* **103**, 1317-1324 (2001).
13. Zankov, D.P., Ding, W.G., Matsuura, H. & Horie, M. Open-state unblock characterizes acute inhibition of I potassium current by amiodarone in guinea pig ventricular myocytes. *J Cardiovasc Electrophysiol* **16**, 314-322 (2005).
14. Gray, D.F., *et al.* Amiodarone inhibits the Na(+)-K+ pump in rabbit cardiac myocytes after acute and chronic treatment. *The Journal of pharmacology and experimental therapeutics* **284**, 75-82 (1998).
15. Watanabe, Y. & Kimura, J. Acute inhibitory effect of dronedarone, a noniodinated benzofuran analogue of amiodarone, on Na+/Ca2+ exchange current in guinea pig cardiac ventricular myocytes. *Naunyn-Schmiedeberg's archives of pharmacology* **377**, 371-376 (2008).
